# Polymorphism of the *GLIS3* gene in a Caucasian population and among individuals with carbohydrate metabolism disorders in Russia

**DOI:** 10.1186/s13104-018-3338-1

**Published:** 2018-04-02

**Authors:** E. V. Shakhtshneider, S. V. Mikhailova, D. E. Ivanoshchuk, P. S. Orlov, A. K. Ovsyannikova, O. D. Rymar, Yu. I. Ragino, M. I. Voevoda

**Affiliations:** 1 Research Institute of Internal and Preventive Medicine–Branch of Institute of Cytology and Genetics, Siberian Branch of Russian Academy of Sciences, Bogatkova Str. 175/1, Novosibirsk, 630004 Russia; 2 Novosibirsk State University, Pirogova Str. 1, Novosibirsk, 630090 Russia; 3 Federal Research Center Institute of Cytology and Genetics, Siberian Branch of Russian Academy of Sciences, Prospekt Lavrentyeva 10, Novosibirsk, 630090 Russia

**Keywords:** Maturity onset diabetes of the young, MODY, Type 2 diabetes mellitus, GLIS3, rs806052, rs143051164, rs149840771, Single-nucleotide polymorphism, Population

## Abstract

**Objective:**

Earlier, GLIS3 gene polymorphisms have been shown to be associated with the development of maturity onset diabetes of the young (MODY). We screened *GLIS3* gene sequences among patients with MODY to identify probably pathogenic variants by whole-exome sequencing. We estimated frequency of rare single-nucleotide variants in the coding region of *GLIS3* in a Caucasian population and among individuals with carbohydrate metabolism disorders in Russia.

**Results:**

We identified 15 single-nucleotide variants in *GLIS3*. Three rare variants (minor allele frequency < 1%) rs806052, rs143051164, and rs149840771 were genotyped in 126 cases of MODY, in 188 patients with type 2 diabetes mellitus (DM2), and 564 randomly selected Caucasian individuals in Russia. A heterozygous rs806052 variant was identified in one patient with DM2; c.1270T frequency was 0.003. Prevalence of rs143051164 c.844G was 0.003 in the control population and 0.004 and 0.003 in MODY and DM2 samples, respectively. Prevalence of rs149840771 c.2096A was 0.003 and 0.004 in the control population and among MODY patients, respectively. In DM2 patients, rs149840771 c.2096A was not identified. We did not detect any associations of rs806052, rs143051164, and rs149840771 with carbohydrate metabolism disorders among patients with MODY and DM2 in Russia.

**Electronic supplementary material:**

The online version of this article (10.1186/s13104-018-3338-1) contains supplementary material, which is available to authorized users.

## Introduction

Maturity onset diabetes of the young (MODY) is a hereditary form of diabetes with autosomal dominant inheritance and is characterized by onset at a young age and the presence of an initial defect of pancreatic β-cell function. This type of diabetes differs from classic types of diabetes mellitus—type 1 (DM1) and type 2 (DM2)—in disease progression, in treatment strategies, and prognosis [1]. To date, 13 types of MODY (MODY1 through MODY13) have been identified, each associated with mutations in a specific gene: *HNF4A*, *GCK*, *HNF1A*, *PDX1*, *HNF1B*, *NEUROD1*, *KLF11*, *CEL*, *PAX4*, *INS*, *BLK*, *KCNJ11*, and *ABCC8* [2]. Dysfunction of other genes causes 11–30% of cases of MODY and are defined as MODY-X [3]. Previously, some of *GLIS3* gene polymorphisms have been shown to be associated with the development of MODY [4].

The gene codes for a Krüppel-like zinc finger transcription factor that is located in a nuclear compartment of the cell and in the Golgi complex. GLIS3 expression in the embryo shows spatial and temporal dependence on embryogenesis stages, whereas in adults it is tissue-specific and depends on cell type. GLIS3 expression occurs mainly in kidneys and in the pancreas, thyroid, thymus, uterus, ovaries, brain, and lungs [5].

GLIS3 plays a critical role in the regulation of many physiological processes (insulin gene expression, β-cell formation, thyroid hormone biosynthesis, spermatogenesis, and kidney function) [6]. It is one of the most differentially expressed genes in β-cells between healthy people and patients with DM2 [7]. *GLIS3* mRNA expression is decreased by 50% in pancreatic islets of patients with DM2 in comparison with healthy people [8]. It is known that mutations in GLIS3-binding sites of the *INS* gene promoter cause neonatal diabetes [9]. All single-nucleotide variants (SNVs) of *GLIS3* that have been associated with DM1 and DM2 in genome-wide association studies are located in noncoding regions in contrast to the SNVs associated with neonatal diabetes. It is thought that these substitutions may regulate *GLIS3* gene expression. Probably, they are associated with β-cells’ ability to respond to immune, viral, and metabolic stressors and have made the *GLIS3* gene a common member of the genetic risk factors of DM1 and DM2 [10].

The aim of this study was identification of probably pathogenic variants of *GLIS3* in patients with MODY using a whole-exome sequencing approach and estimation of the frequency of rs806052, rs143051164, and rs149840771 in a Russian population and DM2 patients.

## Main text

### Methods

The study protocol was approved by the local Ethics Committee of the Institute of Internal and Preventive Medicine (a branch of the Institute of Cytology and Genetics, the Siberian Branch of the Russian Academy of Sciences, Novosibirsk, Russia). Written informed consent to be examined and to participate in the study was obtained from each patient. For individuals younger than 18 years, the informed consent was signed by a parent or legal guardian.

The inclusion criteria were as follows: signing informed consent for participation in the study, a verified diagnosis of diabetes, a debut of the disease for probands at the age of 35 years and earlier, the absence of obesity, absence of pancreas islet cell antibodies, absence of glutamic acid decarboxylase antibodies, sufficient secretion of β-cells, absence of necessity of insulin therapy, and the absence of ketoacidosis at the onset of the disease. The following exclusion criteria were applied: a history of tuberculosis of lungs or other organs or of infection with the human immunodeficiency virus, an infectious disease caused by a hepatitis B virus or a hepatitis C virus that requires antiviral treatment, substance abuse or alcoholism for 2 years before the examination.

In the first phase of the study, whole-exome sequencing was performed on 21 probands with a MODY phenotype and five of their first-degree relatives (three with a MODY phenotype) for analysis of the *GLIS3* mutation spectrum. A total of 26 patients underwent a full medical examination; a biochemical blood test; determination of HbA1c, C-peptide, anti-GAD (glutamic acid decarboxylase) antibodies, pancreas islet cell antibodies (ICA), thyroid status, and microalbuminuria; abdominal and renal ultrasonography; real-time continuous glucose monitoring with Medtronic Paradigm MMT-722; and genetic tests. Genomic DNA was isolated from leukocytes of venous blood by phenol–chloroform extraction [11]. Quality of the extracted DNA was assessed on a capillary electrophoresis system (Agilent 2100 Bioanalyzer; Agilent Technologies Inc., USA). Sequencing of the first group of samples (11 patients) was conducted on an Ion Proton instrument (Thermo Fisher Scientific). Whole-exome libraries were prepared with the AmpliSeq Exome Kit (Thermo Fisher Scientific). The data-processing pipeline included mapping the reads to the reference human genome (GRCh37) using the Burrows–Wheeler Aligner (BWA) v.0.7.12 software followed by SNV calling performed by means of GATK. SNV annotation was conducted in the IonReporter 4.0 software. The average depth of coverage was 30×. Sequencing in the second group of patients (15 individuals, respectively) was carried out on an Illumina HiSeq 1500 instrument (Illumina, San Diego, CA, USA). The enrichment and library preparation were performed using the SureSelectXT Human All Exon V5 + UTRs Kit. Reads were mapped to the reference human genome (GRCh37) in the BWA v.0.7.12 software. PCR duplicates were removed by means of the Picard software. A search for polymorphisms was conducted using the Genome Analysis Toolkit v.3.3 package by the procedure for local remapping of short insertions/deletions and recalibration of the read quality. The depth of coverage was from 34× to 53×.

In sequenced groups, we filtered sequence variants if they were present in 10 or more variant reads with a quality score ≥ 30. In this study, the search for polymorphic sites covered known MODY genes and *GLIS3*. A comparison of the detected substitutions was performed with a number of specialized databases: the 1000 Genomes Project (http://www.1000genomes.org/), dbSNP database (https://www.ncbi.nlm.nih.gov/SNP/), and ClinVar (http://clinvar.com/). We selected the spectrum of rare sequence variants in MODY genes (nonsynonymous, alternative splice site, and coding indels variants) and only nonsynonymous SNVs for common substitutions. Rare variants were selected if their minor allele frequency (MAF) was ≤ 1% in the 1000 Genomes database and if they were present in the dbSNP database.

The possible functional and significant effects of SNVs were assessed by means of in silico tools PolyPhen-2 v2.2.5 (http://genetics.bwh.harvard.edu/pph2/dokuwiki/about), SIFT (http://sift.jcvi.org/), and PROVEAN (http://provean.jcvi.org/index.php). Web services PolyPhen-2 v.2.2.5 and Provean/SIFT predict a possible impact of an amino acid substitution on the structure and function of a human protein.

In the second phase of analysis, we selected only the functional variants with MAF < 0.5% and a potential pathogenic effect: rs806052, rs143051164, and rs149840771. In most cases, the MODY-causing SNVs are rare variants with low MAFs and cannot be highly distributed in the population; therefore, we focused on these substitutions. Verification for rs806052, rs143051164, and rs149840771 was performed by Sanger sequencing and oligonucleotides primers for SNVs were designed in the Primer-Blast software (https://www.ncbi.nlm.nih.gov/tools/primer-blast/). The oligonucleotides are shown in Additional file 1. These SNVs were tested by real-time PCR (Additional file 1) in 564 randomly selected individuals (Caucasians in Russia), in 188 patients with DM2, and in 126 patients with a MODY phenotype (Table 1).

**Table 1:** Demographic characteristics of the tested individuals

	Caucasian population	Type 2 diabetes mellitus	MODY
Number	564	188	126
Age, years	45–69	45–69	1–38
Mean age ± SD, years	54.2 ± 0.4	59.0 ± 6.7	23.8 ± 2.5

*SD* standard deviation

The population group included in the analysis was selected from a survey of the population interviewed within the framework of the HAPIEE project [12], Novosibirsk, Russia (9360 participants, aged 45–69 years, men 50%, Caucasians 97%). A total of 564 randomly selected patients (some may have diabetes, age 54.2 ± 0.4, mean ± SD) were included.

The DM2 group consisted of 68 women and 87 men (age 59.0 ± 6.7, mean BMI among men: 31, women: 32). The DM2 sample was randomly selected from the HAPIEE participants [12]. We used the criteria of the American Diabetes Association [13] for the diagnosis of DM2.

A total of 126 patients with a MODY phenotype (age 23.8 ± 2.6) underwent the same medical examination as described above.

Rs806052 was genotyped by the TaqMan SNP assay (Synthol, Russia) and StepOnePlus 7900HT Real-Time PCR System (Thermo Fisher Scientific, USA). Rs143051164 and rs149840771 were genotyped by the TaqMan SNP assay (BioLabMix, Russia) and StepOnePlus 7900HT Real-Time PCR System (Thermo Fisher Scientific, USA).

Statistical data processing was performed in the IBM SPSS Statistics 23.0 software. Validity of differences in allele prevalence between groups was determined using the χ^2^ criterion. Differences were considered statistically significant at p < 0.05.

### Results

The spectrum of *GLIS3* gene polymorphism in patients with a MODY phenotype was identified by the whole-exome sequencing method. During this analysis, 15 SNV were found (Table 2). Most of these SNVs are common variants and located in noncoding exon–intron boundaries. Rs806052 is located in a DNA-binding domain, rs143051164 in the region of potential interaction with proteins ISL1, ITCH, Smurf 2, Smurf 1, DNAJA1, and HIPK3 [14], and rs149840771 is located near the transactivation C-terminal domain of the GLIS3 protein.

**Table 2:** The *GLIS3* gene polymorphism spectrum in patients with a MODY phenotype in Russia

Chromosome: position	Localization	aa or nucleotide changes	SNV	Zygosity	Clinical significance (ClinVar)	MAF from 1000G
chr9:3828379	Exon 11	p.Leu896Phe/c.2686C>T	rs76094493	Heterozygous	Likely benign	A = 0.04
chr9:3829212	Intron 10	T>C	rs7029652	Heterozygous	NA	C = 0.14
chr9:3855963	Intron 9	delA	rs397766987	Heterozygous	NA	A = 0.36
chr9:3879646	Intron 7	G>A	rs17692969	Heterozygous	NA	A = 0.026
chr9:3879693	Intron 7	T>C	rs4740742	Heterozygous		C = 0.09
chr9:3898723	Exon 6	p.Arg699His/c.2096G>A	rs149840771	Heterozygous	NA	T = 0.0002
chr9:3932283	Intron 6	C>G	rs535978	Heterozygous, homozygous GG	NA	G = 0.40
chr9:3932521	Intron 5	C>T	rs587571	Heterozygous	NA	T = 0.04
chr9:3937255	Intron 4	T>A	rs676935	Heterozygous	NA	A = 0.37
chr9:3937288	Intron 4	G>A	rs10974212	Heterozygous	NA	A = 0.32
chr9:4118111	Exon 4	p.Pro456Gln/c.1367C>A	rs6415788	Homozygous AA	Other	G = 0.32
chr9:4118208	Exon 4	p.Ser424Pro/c.1270T>C	rs806052	Homozygous CC	Other	A = 0.001
chr9:4118634	Exon 4	p.Pro282Ala/c.844C>G	rs143051164	Heterozygous	Uncertain significance allele	C = 0.0004
chr9:4285986	Intron 2	G>C	rs10758591	Heterozygous	NA	C = 0.34
chr9:4298537	Intron 1	G>A	rs12340657	Heterozygous	NA	A = 0.23

*NA* not available, *MAF* minor allele frequency

The GG rs806052 (c.1270T>C) genotype in the homozygous form was identified in our group of Caucasians in Russia and in all patients with MODY (Table 3). This variant is present with a rare allele in one patient with MODY, but after validation it was not confirmed. Nevertheless, we tested this SNV in our groups because it had been found in patients with DM2 and seemed interesting [15]. In the DM2 group, a heterozygous rs806052 variant was identified in one male patient without a family history of diabetes mellitus. Prevalence rates of rs806052, rs143051164, and rs149840771 are presented in Table 3.

**Table 3:** Prevalence of rs806052, rs143051164, and rs149840771 in the tested samples

Genotypes	Caucasian population	MODY	Type 2 diabetes mellitus
rs806052			
GG, % (n)	100 (476)	100 (96)	99.7 (171)
AG, % (n)	0 (0)	0 (0)	0.3 (1)
AA, % (n)	0 (0)	0 (0)	0 (0)
rs143051164			
GG, % (n)	99.7 (561)	99.6 (125)	99.7 (187)
GC, % (n)	0.3 (3)	0.4 (1)	0.3 (1)
CC, % (n)	0 (0)	0 (0)	0 (0)
rs149840771			
CC, % (n)	99.7 (560)	99.6 (125)	100 (188)
CT, % (n)	0.3 (4)	0.4 (1)	0 (0)
TT, % (n)	0 (0)	0 (0)	0 (0)

Frequencies of rs806052, rs143051164, and rs149840771 in the Russian population are close to the ones described in databases.

Prevalence of rare allele C rs143051164 (c.844C>G) in the Caucasian population was 0.003, in the MODY group 0.004, and in the DM2 group 0.003. In the Caucasian group, a heterozygous genotype was identified in three individuals without carbohydrate metabolism disorders and without a family history of diabetes mellitus.

Prevalence of rare allele T rs149840771 (c.2096G>A) in the Caucasian population was 0.003, and in the MODY group, 0.004. In the DM2 group, the rare allele of rs149840771 was not identified.

### Discussion

A number of studies have shown that *GLIS3* plays a role in the regulation of at least four different processes associated with development and normal functioning of the pancreas. Regulation of differentiation of pancreatic islets at an early stage of embryogenesis via activation of transcription factor neurogenin 3 (Ngn3) is involved in β-cell development. Impairment of this process leads to neonatal diabetes. Control of β-cell proliferation under stressful conditions in adults by the regulation of cyclin D2 (CCND2) expression is important for a compensatory increase in β-cell mass during obesity. Impairment of this regulatory step causes the development of DM2. Two more processes—β-cell apoptosis regulation by activation of SRp55 splicing factor expression and regulation of insulin gene expression—can perform a major function at all stages of organismal development [16].

There are more than 10 genes, and many variants of these genes have been found to be associated with diabetes phenotypes. These data allowed us to suppose that substitutions in exons of *GLIS3* are associated with MODY-X or are phenotype modifiers in DM2.

No differences were identified in allele frequencies of the analyzed SNV of GLIS3 in patients with confirmed MODY and the other two groups (patients with DM2 and the Caucasian population). We did not find any novel mutation in coding and splicing site regions of the *GLIS3* gene.

Considering the lack of data on a correlation between rare exon SNVs rs806052, rs143051164, and rs149840771 of the GLIS3 gene and the development of MODY and DM2 in this study, we suppose that these variants are not associated with these pathological phenotypes in Russia. It seems reasonable to analyze a noncoding part of this gene by searching for SNVs associated with different forms of diabetes mellitus.

## Limitations

The *GLIS3* gene mutation spectrum did not show substantial differences among the different tested samples. Whole-exome sequencing did not reveal new SNVs in *GLIS3* of patients with MODY in Russia.

The results of genotyping of the Caucasian group and patients with carbohydrate metabolism disorders allow us to suggest that rare SNVs rs806052, rs143051164, and rs149840771 of the *GLIS3* gene do not take part in the pathogenesis of MODY and DM2 in Russia. One of the limitations of our study is the low sample size. Another limitation is analysis of SNVs only in the coding region of *GLIS3* because there are some SNVs in the noncoding region that are associated with DM1 and DM2 [10].

## Additional file

**Additional file 1.** Genetic analysis of rs806052, rs143051164, and rs149840771. Sequencing, RT-PCR conditions, and in silico analysis for the GLIS3 gene.

## Abbreviations

BWA: Burrows–Wheeler Aligner
DM: diabetes mellitus
DM1: type 1 diabetes mellitus
DM2: type 2 diabetes mellitus
DNA: deoxyribonucleic acid
GAD: glutamic acid decarboxylase
GATK: Genome Analysis Tool Kit
GLIS3: GLI-similar zinc finger
HbA1c: glycated hemoglobin
ICA: pancreas islet cell antibodies
MODY: maturity onset diabetes of the young
mRNA: messenger ribonucleic acid
NGS: next-generation sequencing
PCR: polymerase chain reaction
SNV: single-nucleotide variant
UTR: untranslated region
