# Supplementary material for: Polymorphism of the GLIS3 gene in a Caucasian population and among individuals with carbohydrate metabolism disorders in Russia
Source: BMC Res Notes. 2018 Apr 2;11:211. doi: 10.1186/s13104-018-3338-1 (PMC5880065; doi:10.1186/s13104-018-3338-1)
Supplement: Supplementary file 1 — Additional file 1. Genetic analysis of rs806052, rs143051164, and rs149840771. Sequencing, RT-PCR conditions, and in silico analysis for the GLIS3 gene. [file 13104_2018_3338_MOESM1_ESM.docx]

**Genetic Analysis of rs806052, rs143051164, and rs149840771**

The verification of rs806052, rs143051164, and rs149840771 was performed by Sanger sequencing, and the oligonucleotides primers for SNVs were designed in the Primer-Blast software (<https://www.ncbi.nlm.nih.gov/tools/primer-blast/>). The oligonucleotides and reaction conditions are shown in Additional Table 1. PCRs were set up using BioMaster LR HS-PCR (2×) (BioLabMix, Russia) 1 μl of each primer and 1 μl of DNA, with a total final volume of 25 μl. The thermocycling programs for rs143051164 and rs149840771 consisted of initial denaturation at 94 °C for 3 minutes, and then 35 cycles at 94 °C for 30 seconds, annealing temperature for 30 seconds (Additional Table 1), and 72 °C for 50 seconds. The thermocycling program for rs806052 consisted of initial denaturation at 94 °C for 3 minutes, and then 30 cycles at 94 °C for 20 seconds, 60 °C for 20 seconds (Additional Table 1), and 72 °C for 40 seconds. The PCR products were evaluated by electrophoresis in a 5% polyacrylamide gel after visualization with an ethidium bromide solution. A 100-bp DNA ladder (SibEnzyme, Russia) was run on each gel as molecular size markers. The amplicons were purified using Agencourt AMPure Xp beads (Beckman Coulter, USA). The sequencing reactions were carried out on an automated ABI 3500 DNA sequencer (Thermo Fisher Scientific, USA) with the BigDye Terminator v3.1 Cycle Sequencing Kit (Thermo Fisher Scientific, USA). The sequences were analyzed in the Vector NTI® Advance software (Thermo Fisher Scientific). The hg19 version of the human genome served as a reference sequence for the alignment. The possible functional and significant effects of *GLIS3* missense variants were predicted by means of *in silico* tools PolyPhen-2 v2.2.5 (http://genetics.bwh.harvard.edu/pph2/dokuwiki/about), SIFT (http://sift.jcvi.org/), and PROVEAN (http://provean.jcvi.org/index.php).. The Web services PolyPhen-2 v.2.2.5 and Provean/SIFT predict a possible impact of an amino acid substitution on the structure and function of a human protein. For rs806052 and rs149840771, *in silico* analysis predicted a neutral effect both in PolyPhen-2 and in Provean/SIFT. For rs143051164, PolyPhen-2 and SIFT predicted a possibly damaging effect with a score of 0.649 (sensitivity: 0.87; specificity: 0.91) and score of 0.039, respectively.

The analysis was performed using commercial TaqMan SNP assays. Rs806052 was genotyped by the TaqMan SNP assay and the PCR Master Mix (Synthol, Russia) on a StepOnePlus 7900НT Real-Time PCR System (Thermo Fisher Scientific, USA). Rs143051164 and rs149840771 were genotyped by the TaqMan SNP assay and PCR Master Mix BioMaster HS-qPCR Hi-ROX (2×) (BioLabMix, Russia) on the StepOnePlus 7900НT Real-Time PCR System (Thermo Fisher Scientific, USA).

Additional Table S1. Primers used for sequencing of the *GLIS3* gene

| SNV | Forward  (5′→3′) | Reverse  (5′→3′) | Annealing temperature, °C | Product size, b.p. |
| --- | --- | --- | --- | --- |
| rs806052 | GCATGCAACAGCTGGAGC | AGGAGGGAGCGGAGGC | 60 | 173 |
| rs143051164 | TCGGCCTCAAGCATGAAGCA | GCGACGTGCGGATGATGGTA | 62 | 316 |
| rs149840771 | GGACCGGCTCTCCTTATCGA | GTGGAGAGCAATTTGCAGCC | 60 | 332 |

b.p. - base pair
